# Supplementary material for: A webcam-based machine learning approach for three-dimensional range of motion evaluation
Source: PLoS One. 2023 Oct 23;18(10):e0293178. doi: 10.1371/journal.pone.0293178 (PMC10593217; doi:10.1371/journal.pone.0293178)
Supplement: S1 Appendix — (DOCX) [file pone.0293178.s001.docx]

# Appendix

**Joint Acronym Lookup**

| Acronym | Joint Name |
| --- | --- |
| **Head** |  |
| LFHD/LFHD | Left/Right Front of the Head |
| LBHD/LBHD | Left/Right Back of the Head |
| LEAR/REAR | Left/Right Ear |
| NOSE | Nose |
| **Torso** |  |
| LSHO/RSHO | Left/Right Shoulder |
| LASI/RASI | Left/Right Anterior Sacroiliac |
| LPSI/RPSI | Left/Right Posterior Sacroiliac |
| **Upper Limbs** |  |
| LELB/RELB | Left/Right Elbow |
| LWRI/RWRI | Left/Right Wrist |
| LWRA/RWRA | Left/Right Wrist A (Anterior) |
| LWRB/RWRB | Left/Right Wrist B (Posterior) |
| **Lower Limbs** |  |
| LHIP/RHIP | Left/Right Hip |
| LKNE/RKNE | Left/Right Knee |
